# India and its pluralistic health system – a new philosophy for Universal Health Coverage

**DOI:** 10.1016/j.lansea.2022.100136

**Published:** 2022-12-31

**Authors:** Sarika Chaturvedi, John Porter, Geetha Krishnan Gopalakrishna Pillai, Leena Abraham, Darshan Shankar, Bhushan Patwardhan

**Affiliations:** a Directorate of Research, Dr D Y Patil Vidyapeeth, Pune, India; b Clinical Research and Global Health and Development, London School of Hygiene and Tropical Medicine, London, UK; c Traditional, Complementary, and Integrative Medicine Unit, Integrated Health Service Department, World Health Organization, Geneva, Switzerland; d Centre for Studies in Sociology of Education, Tata Institute of Social Sciences, Mumbai, India; e Transdisciplinary University of Health Sciences and Technology, Bengaluru, India; f Centre for Complementary and Integrative Health, Interdisciplinary School of Health Sciences, Savitribai Phule Pune University, Pune, India

**Keywords:** Traditional medicine, Public health, Health policy

## Abstract

In this article we attempt to put forth insights into using traditional medicine (TM) systems to achieve Universal Health Coverage (UHC). We discuss the need for reimagining India's health system and the importance of an inclusive approach for UHC. We comprehend the challenges with appropriate use of TM systems and the lessons from international experience of integrating TM systems. We highlight the pathways for better utilization of TM systems for UHC in India.

In the quest to evolve better and more resilient global health systems, increasing attention is being directed to the roles played and contributions made by the indigenous sources of knowledge, including the Traditional and Complementary Medicine Systems.^1^ Undoubtedly, there is growing awareness of the fact that traditional knowledge systems can definitely contribute to higher acceptability and coverage among the population rather than relying solely on one system.^2^ According to the World Health Organization (WHO) 88 per cent of all countries are estimated to use traditional medicine and about 40 per cent of existing pharmaceutical formulations are based on natural products.^3^

Bridging the gaps between Western and indigenous knowledge systems is now considered an important imperative for improved health research and outcomes.^4^ India is known for the popular use of traditional medicine (TM) systems — Ayurveda, Yoga, Unani, Siddha, and Sowa-Rigpa, that along with Homeopathy, are now termed as the AYUSH systems. Since India's independence, experts and practitioners have grappled with the issue of medical pluralism and the poor integration of these traditional systems into the biomedical mainstream. While historically these systems have been sidelined,^5^ the AYUSH sector is now more functional and growing exponentially with an annual turnover of $18 billion with services to millions of citizens.^6^

A cross-sectorial effort to reform India's health system is underway through the Lancet Citizen's Commission to Reimagine India's Health System. One of the multiple efforts of the Commission is to identify ways to address the challenges to achieving Universal Health Coverage (UHC) by exploring the role of TM systems.^7^ UHC implies that all people have access to the health services they need, when and where they need them, without financial hardship. It includes the full range of essential health services, from health promotion to prevention, treatment, rehabilitation, and palliative care.^8^

## Reimagining India's health system

The current dominance of biomedicine in the health systems of countries like India, which are home to the long-standing TM systems, is rooted in their colonial histories. The structure, philosophy, and perspectives held within the biomedical model, come from the colonizing era of European history, which was focused on conquest and control. The dominant aspect of health must be altered so as to become inclusive of the local and indigenous perspectives, that prevailed before the colonial period. The “Decolonization of health science knowledge systems” is long overdue. Reimagining the health system for UHC will help to realign the current focus on biomedicine. It will create roadmaps to inclusive and pluralistic health systems that are connected to the health of the ecosystem and the needs of the population.

## Importance of a balanced and inclusive approach for UHC

Biomedicine is human-centric and incognisant of our relationship and deep connection with nature and the planet. The Indian health system respects and honours pluralism and the different perspectives and insights into health as provided by indigenous communities through their knowledge of life, health, balance, and nature.

For UHC to be effective a balance between individual and systemic levels is essential. In the current context of the health system in India, the family or household level is not adequately considered — the current infrastructure includes clinics and hospitals but not the household and community; and the drugs and supplies considered are only from pharmaceutical preparations but not from plants and other local resources used by several sections of the population.^9^ It is possible to attain a more inclusive, balanced, and pluralistic national system that includes and honours individuals and their capacities for self-care as an important contribution to the overall health of the population. Such a health system will provide excellent coverage for AYUSH as well as for biomedical consultation and treatment. This concept of “population self-reliance” in health is referred to as the fourth-tier.^9^ This respects the people's capacity for self-care and their responsibility to the health system and its values. The fourth-tier concept recognizes an individual's ability for self-care at home and therefore for self-reliance in health. Self-care is when people apply their traditional and local health knowledge and follow appropriate or related practices to maintain their health without always relying on, or needing institutional support. This concept is rooted in the perspectives of health in the Ayurveda and Yoga systems.

The current health-system model needs to recognize the role of local epistemic expertise, the local forms of care, and the contributions these communities can make to the overall population and economic health of the system.^10^ The human values of the dignity of the individual, respect for others, and trust in the community are integral to the philosophies of TM; they are important for a health system that appreciates the holistic nature of life and our connection to the ecosystem. These values, however, are currently submerged.^11^ We need a balance between evidence and values, to enhance acceptability. The approaches contained within the traditional knowledge systems are important for a balanced and inclusive health system within the philosophy of UHC.

## People's preferences matter to the UHC system

Health-seeking behaviour in India is complex and is influenced by ideological and material factors. Literature reveals that people follow pluralistic practices.^12^ Studies conducted in the1950s concluded that people choose home remedies and follow plural systems of medicine because of the unavailability or inaccessibility of biomedical care.^13^ It has been popularly assumed that these choices and behaviours are due to ignorance or illiteracy.^12^ Notably, despite the increased availability and accessibility of biomedical services, both in the public and private sectors and across rural and urban areas and social classes, this health-seeking behaviour persists.^13^ The preference for home remedies, especially to address common and seasonal illnesses, is due to their easy access and low cost. Efficacy and costs are important criteria in people's choice of medical systems, especially for chronic ailments. Costs apart, the growing concern about the side-effects of biomedical treatments as perceived or experienced is certainly a deterrent. Therefore, people's diverse preferences are governed by a complex combination of factors at the individual, sociocultural, and health-system levels.

Medical pluralism exists in other parts of the world as well; however, it is often with one dominant system and others are considered ‘alternative’. In India, all six of the AYUSH systems are not only legitimized by the State but their knowledge and practice are ingrained in the culture of society, to the extent that some of the interventions from the ancient medical systems are integral to home-care traditions. For example, oil massage of infants, as the TM systems recommend, is practiced widely at homes disregarding any modern medical advice.^14^ However, in a vast and diverse country like India, the extent to which plural medical knowledge has been culturally incorporated, has been uneven.

## Universalization in the context of diversities

To incorporate regional diversities in the UHC planning, it is important to understand and assess their nature, the specificity of cultural practices, the depth of the existing medical knowledge, and available expertise in a region. Local history greatly influences medical practices and knowledge systems. They are also embedded in the local language and literature. This is evident from the variations in Ayurveda practices across the country and the way in which the Siddha system in Tamil Nadu is closely entwined with the Tamil language and literature. Similarly, although health-care practices of the tribal communities are linked to the local animal and plant resources, they vary across tribes and ecological terrains. It is important, therefore, to acknowledge the historical evolution of these knowledge systems in different regions of India. Further, the diverse needs of the local communities, divided as they are by caste, class, and gender, with unequal access to resources, become important in the planning for UHC. Recognizing this will ensure that the medical systems are appropriately incorporated into UHC planning and local resources are efficiently utilized. Local knowledge has been getting gradually eroded and several cultural practices have been declining due to policies preventing people's access to those resources and by projecting these practices as ‘primitive’. This erosion could deter the UHC from adopting some of these beneficial practices. Diversities do exist within the medical knowledge traditions; the evolution of these systems varies in different regions of India. Recognizing the diversity of traditional knowledge, the availability of resources, and the needs of the population can inform the incorporation of these systems into the planning of the UHC.

The continued disinclination to seriously engage with indigenous and traditional medicine knowledge shows the epistemic injustice towards the rich knowledge systems.^15^ An important step to address this injustice to the TM systems is to promote authors from the regions where these epistemologies are rooted, such as Asia and Africa. Such authors must be encouraged to contribute tangibly to the synthesis of global knowledge.

## Evidence is not necessarily from RCTs alone

The belief that there is only one kind of medical science is a myth. Ayurveda is considered unscientific because it has a non-molecular, systemic way of observing, classifying, and understanding causality and for mitigating biological change. However, it has a unified theory of bioregulation, with a comprehensive understanding of variability, holistic pharmacology, and pathogenesis. Ayurveda also has a large repository of over 200,000 drug formulations^16^ and effective non-drug interventions such as the *Panchkarma* therapies^17^ that can detoxify impaired physiological processes. This knowledge framework is, theoretically, extremely sophisticated. Nonetheless, the prevalent biomedicine framework dominates through a particular understanding of the concept of ‘evidence’. “Evidence-based medicine” fails to recognize other frameworks, and often categorizes the AYUSH systems as unscientific and lacking evidence. The fact is that biological change can be clinically managed and observed at both the molecular (by biomedicine) and systemic levels (by AYUSH). The TM systems have been practiced for millennia; however, mechanisms for generating evidence from practice are currently lacking and perhaps should be studied and developed further. The present understanding of evidence is not TM-friendly and is often inappropriate for these knowledge systems. Evidence does not necessarily result from experiments alone; real-life usage and outcomes can be important sources as well. Interventions that give no benefit or cause harm cannot remain popular. Furthermore, interventions that are said to be effective and safe in rigorous RCTs that are considered to be the “gold standard method”, need to be withdrawn if surveillance reports on use by the population are unfavourable. An intervention or a practice that millions of people find safe and effective is stronger evidence, akin to phase IV in a trial of a new drug when compared to the conventional new evidence generated from small, controlled studies. Research methods for generating new knowledge, including from TM systems, should evolve with the dynamism of science and not remain fixated on evidence from a particular system of medicine or a particular study design.^18^ The epistemic injustice to TM knowledge may have contributed to the benefits of the AYUSH systems remaining unappreciated. The use of Quinoa for treating malaria in Traditional Chinese Medicine (TCM) is a good example of such neglect of TM knowledge. Although the Chinese have used the Quinoa bark for generations to treat febrile illness and malaria and the fact that the relevant studies have been published in leading Chinese journals over the years, WHO supported its use only in 2006 and brought it to the awareness of the international community.^15^ For several beneficial AYUSH interventions, the situation is similar — investment on research in these systems remains miniscule and the literature produced by Indian authors, even if published in conventional journals, is not taken seriously enough to inspire corrective action.

Innovation, in understanding the mechanisms of generating evidence and acceptance, has become essential. Such innovation has the potential to bring a much-needed change for better integrated health systems. The recent advances in, and availability of, Artificial Intelligence (AI) and Machine Learning (ML) will be useful for providing such evidence. There is growing recognition of the fact that the evidence generated from RCTs and their meta-analysis in the evidence-based medicine approach is not always the best as it emphasizes universality and impersonalization, ignoring the importance of the context in which care is given, the individual variations, and personal preferences.^19^ Research and data are the direct outcomes of investment in research in a particular area. For example, the “lack of evidence” from the AYUSH systems in India is because of gross underinvestment in the sector, but this is often interpreted as the system itself being ineffective or incompetent.

## TM integration-global scenario and lessons for India

Globally, TM systems are available in both high and low-income regions. For example, in China, TCM is already integrated into the mainstream health system.^20^ In the African regions the use of herbal medicine is conspicuous as it also supports local providers.^21^ In Switzerland, a high-income country with world-class biomedical facilities, a legislation governing TM (including Ayurveda) was altered to accommodate the TM practices of the population.^22^ The Malaysian health system allows the practice of other medical systems as does Singapore, although a weakness may exist in the regulatory infrastructure.^1^

The three models of plural medical systems in Southeast Asia, with their varying levels of complementarity/integration as described by Shim^23^, are informative in characterizing the Indian system. These include: the interpenetrative pluralism in China where TM and biomedicine are institutionalized as independent and equivalent systems; the exclusionary model in Korea where the two strictly exclude each other (unlike in China where these practices often crossover each other in practice); and the subjugator model in Japan where TM is subordinate to biomedicine. The Indian model resembles the subjugator model that requires considerable systemic effort to overcome the marginalization of the TM systems, and to allow their potential to be used for the health of the population.

Pluralistic health systems are accepted according to local contexts. They are more readily accepted in societies that have a prevailing TM system; this is in contrast to societies in which the TM systems have evolved and have been ‘medicalized’ into modern medicine. India is at an advantage with several different living health traditions that the people widely practice and use.

## Integrative approach to achieve UHC

Although various models exist for integrating TM systems, it is essential that the local contexts are used to determine the best fit for any region and country.^1^ Integrating the AYUSH systems into mainstream health-care in India has the great potential to improve how the overall health system will perform. The health workforce must understand the need for, and the use of, integrating systems to create a pluralistic health system that satisfies the needs of the population. A dialogue between health providers from different systems is essential so that the best possible outcomes for the patients are achieved. Major reforms are required in Indian medical education to inculcate among medical students and providers the attitude that supports integrating biomedicine and TM. Introducing awareness of the AYUSH systems in school curricula would be an important step to reduce the epistemological barriers in inter-provider communication. Programmes that integrate interventions from different systems are important. With the known strengths and limitations of different systems (including biomedicine), the best options to achieve the desired health goals can be drawn from the different systems. This requires comparative benefit-analysis studies. Existing literature shows the effectiveness of certain TM interventions in chosen conditions. The Ayurveda management of osteoarthritis^24^ for example, is safer and less expensive and yet is not used formally in the health system; there appears to be a preference for relatively less effective and expensive interventions. There are other conditions such as yoga for cardiac rehabilitation,^25^ and Ayurveda for filariasis^26^ where the AYUSH systems either fair better or add value if used together with conventional care. This shows that appropriate policies are needed that are not simply a result of available evidence but are pragmatic decisions that include information on the available expertise and practices. People's wisdom in choosing health system interventions develops over centuries and should not be ignored.

It is important to recognize how technology can deliver TM interventions to people in ways that can increase access and availability. This has the potential to support the self-reliance of the population, as described in the fourth tier.^9^

Financial protection is an important aspect of UHC and a high priority in India, given the high out-of-pocket payments for health services. The relative strength of the AYUSH systems in health-care and their better utilization can save costs on medical care. Economic evaluations are needed to determine the costs and potential gains of using the AYUSH systems. A review of the economic evaluations of homeopathy^27^ has been promising, although more studies are needed for a firm conclusion. Allowing health-care decision-making to be based on cost-effectiveness in addition to clinical efficacy requires promoting economic evaluations in AYUSH interventions.^28^

## Recommendations on the utilization of AYUSH for UHC in India

New research paradigms, models, and strategies are needed to utilize the potential of the AYUSH systems for ensuring UHC in India. The health policy documents in India, such as the National Rural Health Mission (NRHM, 2005), the National Health Mission (NHM, 2013), the National Health Policy (NHP, 2017), and the Ayushmann Bharat Health and Wellness Centres (HWCs, 2020) are integrative of the AYUSH systems and reflect the assumption that a single biomedical system cannot achieve UHC. We recommend the following measures to bring the policies into action:

1. New models of integrative care are required that can cross the boundaries of systems and appropriately utilize the plural systems for UHC. Radical reimagining of the National Health Systems is possible by repositioning the role of both indigenous health sciences and practices, and biomedicine. The strengths of traditional medicine for primary care, disease prevention, health promotion, mental wellbeing, and delaying the progression of chronic diseases need to be utilized along with the advances of modern medicine in critical care, treatment of acute conditions, and diagnostics.

New health research should focus on wellness, bioregulation, general immunity, multi-targeted drugs, and people's ability for self-care. Although research in the fields of economics and health focusing on the AYUSH systems and using a qualitative methodology is important to inform policy, it is currently inadequate. Research to understand the power struggles and tensions between diverse societal groups and knowledge systems is also needed to inform the UHC design in the Indian context of diversity and inequality. Cross-system dialogues at the levels of education, research, and practice are crucial. The areas where AYUSH practices have better and positive results should be further researched in trans-disciplinary frameworks to understand concepts, drugs, therapies, and modes of action in a systems-biology framework. The goal of such research is not merely for the validation of AYUSH, but rather for cross-cultural communication and mutual learning across the worlds of indigenous and Western health sciences.

2. It is important to integrate AYUSH at all three tiers of the health system. In the recent past AYUSH personnel were used to increase the coverage of health services because of the shortage of trained medical staff,^29^ however, this did not increase the efficient availability of AYUSH services.^30^ Utilizing AYUSH personnel must be strategized so as to deliver care in the current system where modern medicine is dominant.

Additionally, executing the fourth tier has to be prioritized so as to materialize the concepts of population self-reliance and the wisdom of the people on health and the health system. This implies promoting the use of beneficial health practices, dietary measures, and lifestyles that the AYUSH systems prescribe and are a part of the local culture.

3. An immediate actionable agenda is to utilize practice-based evidence to reassess clinical evidence and public acceptance of interventions, from both the biomedical and AYUSH systems. An AI-and ML-enabled analysis of big data from 100 reputed clinical establishments from both the systems in the country should be prioritized to generate practice-based evidence that is superior to the information that RCTs can provide. This could help reassign roles to these systems in the national health systems based on real-world data. An important caution in utilizing the AYUSH systems thus is with over-medicalization. Importantly, the holistic nature of the AYUSH systems and considerations of the ecosystem should not be undermined in the efforts at integration. It is essential to understand and be aware that the philosophy of these systems is radically different, and although TM can be integrated into a health system that is currently dominated by a biomedical philosophy, the TM systems hold a broader and holistic philosophy that enfolds biomedicine within it.

## Contributors

Conceptualization — S.C., J.P., G.K., L.A., D.S., B.P.; Writing original draft — S.C.; Writing - review and editing — J.P., B.P. All authors read and approved the final version.

## Declaration of interests

We declare no competing interests.
